# High-Precision Wheat Head Detection Model Based on One-Stage Network and GAN Model

**DOI:** 10.3389/fpls.2022.787852

**Published:** 2022-06-02

**Authors:** Yan Zhang, Manzhou Li, Xiaoxiao Ma, Xiaotong Wu, Yaojun Wang

**Affiliations:** ^1^College of Information and Electrical Engineering, China Agricultural University, Beijing, China; ^2^College of Plant Protection, China Agricultural University, Beijing, China; ^3^College of Economics and Management, China Agricultural University, Beijing, China

**Keywords:** object detection, wheat head, one-stage network, machine learning, generative adversarial network (GAN)

## Abstract

Counting wheat heads is a time-consuming process in agricultural production, which is currently primarily carried out by humans. Manually identifying wheat heads and statistically analyzing the findings has a rigorous requirement for the workforce and is prone to error. With the advancement of machine vision technology, computer vision detection algorithms have made wheat head detection and counting feasible. To accomplish this traditional labor-intensive task and tackle various tricky matters in wheat images, a high-precision wheat head detection model with strong generalizability was presented based on a one-stage network structure. The model's structure was referred to as that of the YOLO network; meanwhile, several modules were added and adjusted in the backbone network. The one-stage backbone network received an attention module and a feature fusion module, and the Loss function was improved. When compared to various other mainstream object detection networks, our model outperforms them, with a *mAP* of 0.688. In addition, an iOS-based intelligent wheat head counting mobile app was created, which could calculate the number of wheat heads in images shot in an agricultural environment in less than a second.

## 1. Introduction

As one of the three grains, wheat, a widely planted cereal crop, is widely planted worldwide. The ear of wheat, known as the wheat head or wheat spike, is a staple food of humans—humans consume most of the wheat head production, and merely approximately one-sixth of it is used for feeding. Wheat can be processed into flour for staple foods or snacks, or fermented into alcohol or biofuel. Wheat's most common growth stages are the green, jointing, heading, filling, and maturity stages. The growth and health statuses of the wheat head have a significant impact on wheat yield and quality from the heading stage to the maturity stage. More specifically, spike number per unit ground area is one of wheat production's most critical agronomic factors. Based on this feature, real-time evaluation can assist in monitoring wheat growth, making management strategies, and then provide an early prediction of the wheat yield. In wheat breeding programs, the wheat head feature can also be selected as a phenotypic trait.

High-precision wheat head recognition is essential for extracting wheat head features and automatically detecting wheat phenotype. Machine vision and deep learning technologies have advanced to the point where the number of wheat heads can theoretically be measured automatically and accurately. Nonetheless, identifying wheat heads *via* machine vision technology is a complicated and tricky task with multiple obstacles:

1. Wheat heads vary dramatically in size, posture, shape, and texture depending on wheat varietals and growth stages. Take wheat heads as an example, their edges' shapes are irregular, and some of their colors are similar to the leaves in particular growth stages.
2. The automatic identification of wheat heads is significantly hampered in the diverse field environment due to mutual shielding between distinct wheat organs and the uneven and unstable natural illumination.
3. Different growth environments for wheat also impact the effect of the detection model. Hence, a machine learning model that can detect wheat heads in various situations with solid generalizability is desperately required.

In the realm of image-based wheat head recognition and other spike-like plant recognition tasks, several researchers have made significant progress. Convolutional neural networks (CNNs) have been widely used in computer vision (such as object detection tasks in this study) due to their powerful feature extraction capabilities. Meanwhile, the particular end-to-end structure allows convolutional neural networks to be trained end-to-end and applied to document recognition; for example, Saleh et al. (2021) proposed a CNN-based model to detect fake news. To automatically distinguish wheat heads based on RGB photos, Tang et al. (2017) and TAN Yun-lan and Ouyang Chun-juan (2019) utilized classic image processing sharpen and smoothing methods such as the Laplacian frequency filter and median filter. The detection accuracy in a test set exceeded 90%, and the detection accuracy in a practical field experiment was greater than that of the artificial wheat head recognition approach. Uddin et al. (2020) utilized a CNN model to examine the number of rice spikes. The model incorporated the feature pyramid network (FPN) (Lin et al., 2017) into the faster region-based CNN network, and the model's accuracy approximated 99%. Allego et al. (2020) proposed an automatic method for wheat heads recognition and counting in digital images captured under realistic fields. The DeepCount method built feature models and fed them into deep CNNs for classification. The suggested method attained the most excellent coefficient of determination (*R*^2^) of 0.89 on an experimental dataset. Grbovi et al. (2019) employed a vehicle camera to collect data samples in a wheat field and used a twin-support-vector-machine segmentation model to train a wheat head detection model. The automatic recognition accuracy of the model was almost identical to that of the manual effect. Fernandez-Gallego et al. (2018) used simple linear clustering to identify wheat heads. The experimental results showed that the recognition accuracy was 94% on a wheat head image set under a high nitrogen application level and 80% on a wheat head image set without nitrogen application. To identify wheat heads, Fernandez-Gallego et al. (2018) employed simple linear clustering. The detection accuracy was 94% on a data set with a high-nitrogen environment and 80% on a data set without nitrogen application.

Additionally, unmanned aerial vehicle (UAV) techniques can help capture wheat head images. Liu et al. (2007) operated UAVs to capture rice head images. They used an improved region-based fully CNN and achieved 87% detection accuracy on their model. Considering UAV's practical values in several situations, especially in the Beyond fifth Generation (B5G), Gopi et al. (2021) suggested a Machine Learning (ML)-assisted algorithm to provide optimal performance during atmospheric disruptions. Alsamhi et al. (2021b) focused on the application of blockchain and Federated Learning (FL) to allow drone edge intelligence for green and sustainable surroundings. They looked into the motivation, structure for intelligent green environments, and integration of FL and blockchain technology. An intelligent technique was also presented for predicting the signal strength from a UAV to Information-of-Things devices in smart cities (Alsamhi et al., 2021a). Because of that, network connectivity can be maintained, appropriate Quality of Service can be provided, and the drone coverage area can be identified. Thanks to the contributions made by previous scientists, UAVs have significantly aided in crop spike detection tasks. Using photos of rice acquired by UAVs, Zhou et al. (2019) employed an unsupervised Bayesian learning algorithm to recognize the rice spike. It achieved 96% in Recall and 72% in Accuracy.

Conclusively, previous research has provided some useful insights into the use of deep learning approaches for wheat head detection. There is still potential for development in terms of detection speed and accuracy and other above-mentioned classical obstacles in this task.

This paper suggested a novel wheat head detection model based on the widely used single-stage object detection network model, YOLO, with the purpose of detecting wheat quickly. The main innovation of the network model proposed in this paper can be summarized in the following points: (1) Add generative sub-network to the attention module to improve the main detection network's performance; (2) Replace the NMS algorithm in the detection network with WBF algorithm; (3) Replace the original *GIoU* calculation in the network by introducing *CIoU* to the loss function.

## 2. Materials and Methods

### 2.1. Data Set and Pre-processing

#### 2.1.1. Data Set

The data set used in this study was retrieved from the Global Wheat Head data set (Kaggle, 2020). Image data were collected and annotated by nine research institutions from seven countries, including Tokyo University, Saskatchewan University, Queensland University, and Nanjing Agricultural University. A number of organizations, including the Global Institute for Food Security, DigitAg, Kubota, and Hiphen, have joined the effort to accurately examine wheat heads.

**Figure 1:**
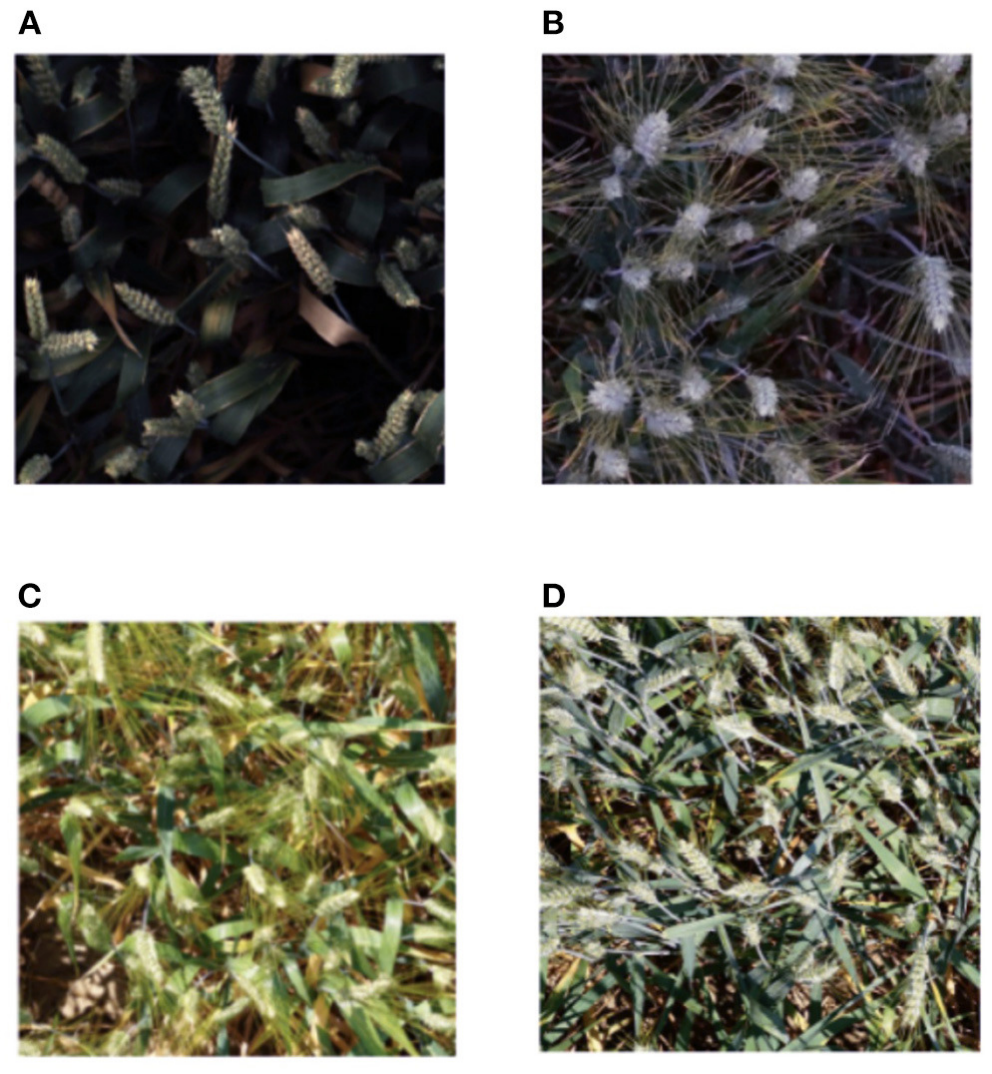
The data set contains pictures taken under various weather conditions, illumination, and growth stages of wheat. **(A)** Photograph taken under shading during the wheat filling stage. **(B)** Photograph taken in half sunshine during the wheat flowering stage. **(C)** Photograph taken in full sunlight during the heading stage of wheat. **(D)** Photograph taken in full sunshine during the flowering stage of wheat.

The data set was divided into two parts, namely, the training set and the test set. The training data set consisted of wheat image data from multiple countries and regions, with more than 3,000 images from Europe (France, UK, Switzerland) and North America (Canada). The test data included about 1,000 photos from China, Australia, and Japan. The data were images of wheat fields with bounding boxes for each identified wheat head. Some of the images did not have labeled wheat sheaf boxes, and the images were recorded in many locations worldwide. Images were captured in a variety of weather situations, illumination, and wheat growth stages, as shown in Figure 1.

#### 2.1.2. Data Set Analysis

Several difficulties were encountered during data pre-processing: (1) wheat in densely planted areas often had overlapping plants in the image; (2) images were blurred when taken under windy conditions; and (3) wheat phenotypes varied with wheat genotypes and growth periods. These are the main challenges to the application of image recognition technology in crop phenotypic analysis.

**Figure 2:**
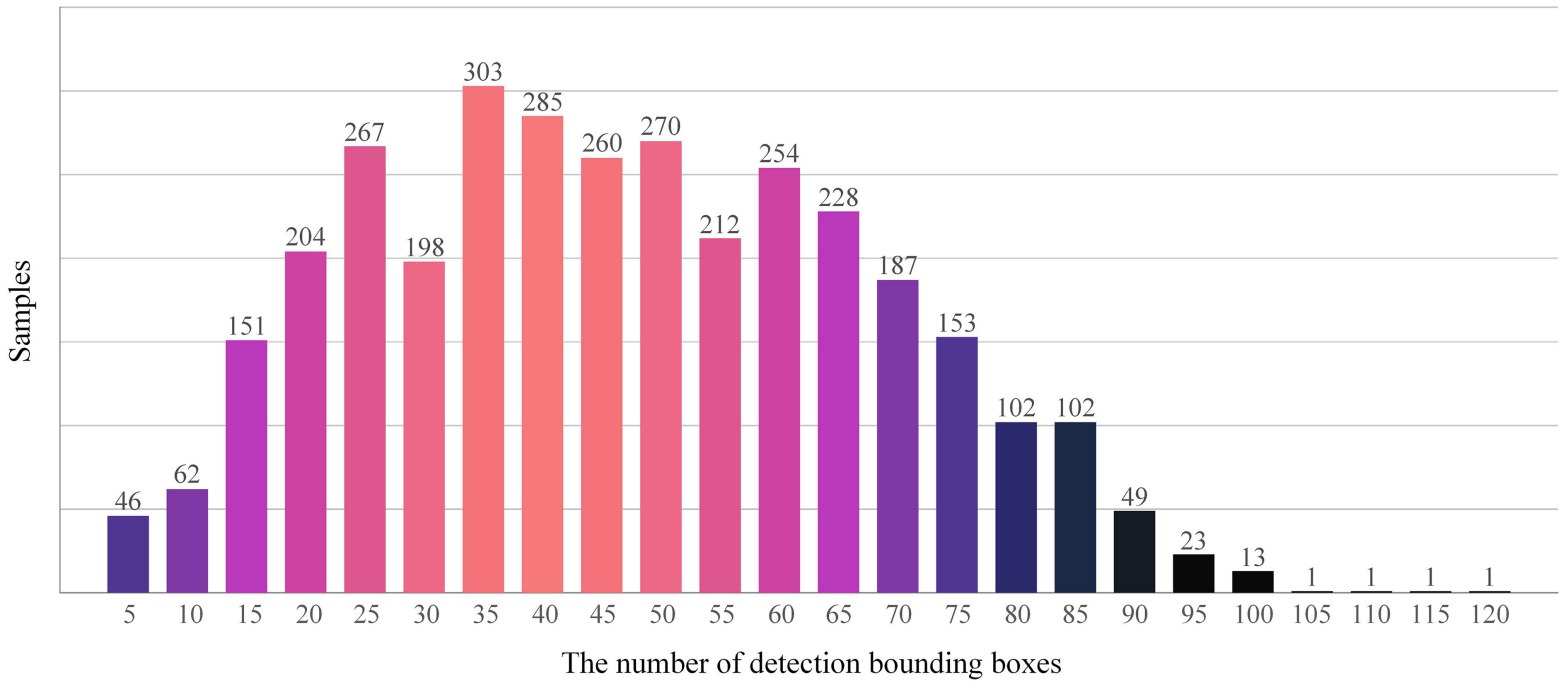
Histogram of the detection boxes in the data set.

**Figure 3:**
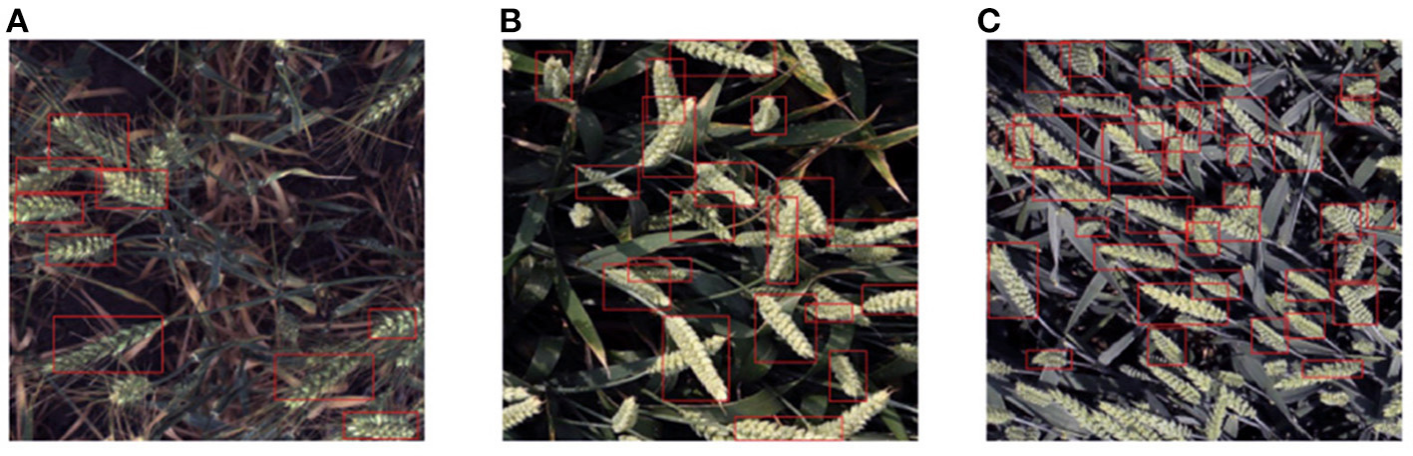
Training samples with different sparsity levels of the detection box number: **(A)** 9 boxes; **(B)** 19 boxes; **(C)** 37 boxes.

As Figure 2 depicts, from the statistical perspective, the number of detection frames in the training set obeyed the normal distribution. The number of detection boxes in most images ranged from 20 to 60, although 49 sample images did not contain detection boxes, and the maximum number of detection boxes in an image was 116. The detection boxes in a single image in the data set may be too sparse or dense, which will make it difficult to train the wheat head detection model. As illustrated in Figure 3, different sparsity degrees of detection box number in sample images of the training data set are offered.

#### 2.1.3. Data Augmentation

The data augmentation method is usually applied in the case of insufficient training samples. If the sample size of the training set is too small, the training of the network model will be insufficient or the model will over-fit. The data amplification method used in this study included two parts: simple amplification and experimental amplification.

**Figure 4:**
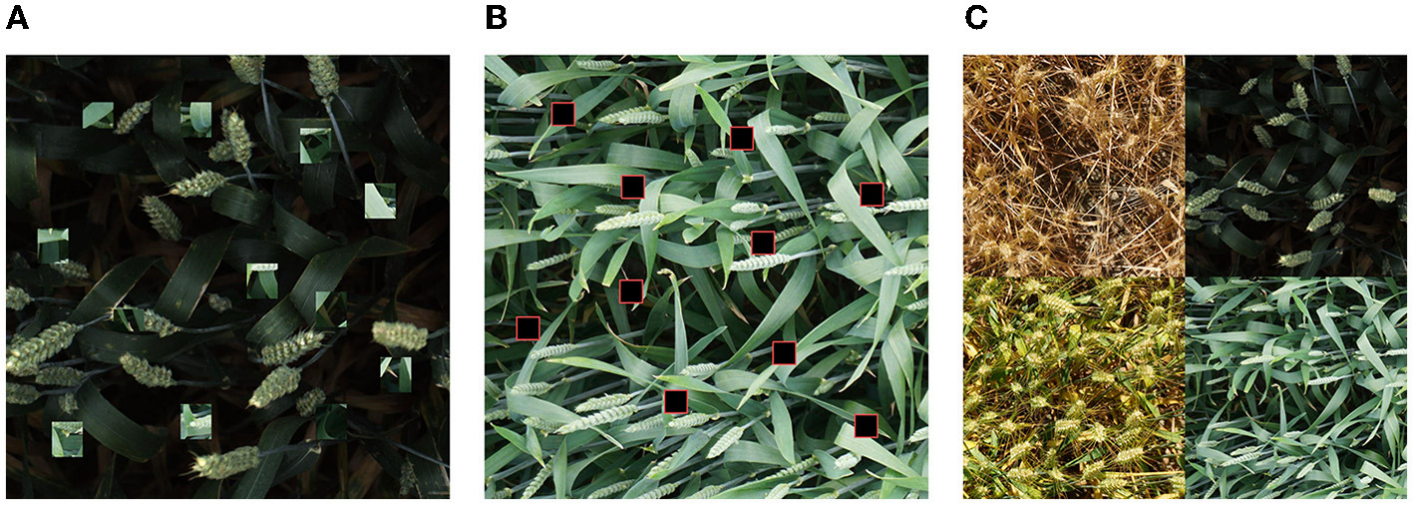
Illustrations of three data enhancement methods: **(A)** CutMix method; **(B)** Cutout method; **(C)** Mosaic method.

1. Simple amplification. Traditional image geometry transformation, including image translation, rotation, cutting, and other operations, can be used for simple data amplification. In this study, the method proposed by Krizhevsky et al. (2012) was adopted. First, each original image was cut into five subgraphs, and then the five subgraphs were flipped horizontally and vertically. The trimmed training set image was counted by outsourcing frames to prevent the part of outsourcing frames from being cut out, and then HSV channel color change was carried out (Sural et al., 2002). In this way, each original image generated 15 extended images. As a result, the training set was expanded from 3,000 image samples to 45,000 data samples.
2. Experimental amplification. Currently, popular data amplification methods in the field of deep learning research include Cutout (DeVries and Taylor, 2017), CutMix (Yun et al., 2019), and Mosaic (Ge et al., 2021). In this study, these three methods were used for further data amplification based on 45,000 training samples. Different amplification methods were used to evaluate the comparative experimental results. The Cutout method randomly cuts out some areas in the sample and fills them with a certain pixel value, and the classification label remains unchanged (Figure 4A). The CutMix method cuts out a part of the area and fills it with the training set randomly instead of 0 pixels. To make full use of the image backgrounds that did not contain wheat heads in the data set, when CutMix was performed, the image with the wheat heads and the image without the wheat heads were subjected to a 1:1 CutMix operation (Figure 4B). The Mosaic method could use multiple pictures at once, and its most significant advantage lies in the fact that it could enrich the background of the detected objects (Figure 4C).

### 2.2. Methods

YOLO (Redmon et al., 2016; Redmon and Farhadi, 2017, 2018; Bochkovskiy et al., 2020), and SSD (Liu et al., 2016) have demonstrated great performance on MS COCO (Lin et al., 2014) and Pascal VOC (Everingham et al., 2010) data sets and are frequently employed in object detection tasks. However, since the anchor parameters of YOLOv5 do not match the actual wheat head data set. The performance of the model obtained by directly training YOLOv5 is not good. The following are the key reasons: Because MS COCO and Pascal VOC data sets are typically used for YOLO and SSD algorithms' training, the algorithm's anchor points are not universal, particularly when it comes to inferior object recognition accuracy. As a result, our high-precision wheat head detection model was presented, which is co-opted for the structure of the YOLOv5 model (Jocher et al., 2022) and is primarily useful for wheat head recognition and is based on the idea of the one-stage network.

Compared with YOLOv5, the main differences of our model follow.

1. An attention module was added in the backbone network to enhance the extraction ability of wheat spike features.
2. Multi-scale feature fusion modules were added to the backbone, and the modules were optimized by referring to the ideas of the feature fusion network FPN and the path aggregation network PANet (Liu et al., 2018).
3. The loss function was improved, and specific loss functions were designed for the recognition modules of wheat spike and background image.
4. The activation function was improved, and the LeakyReLU function, commonly used by CBM modules in the backbone network, was replaced with the Mish activation function.
5. A label smoothing function was added at the output end of the backbone network to prevent classification overfitting.

**Figure 5:**
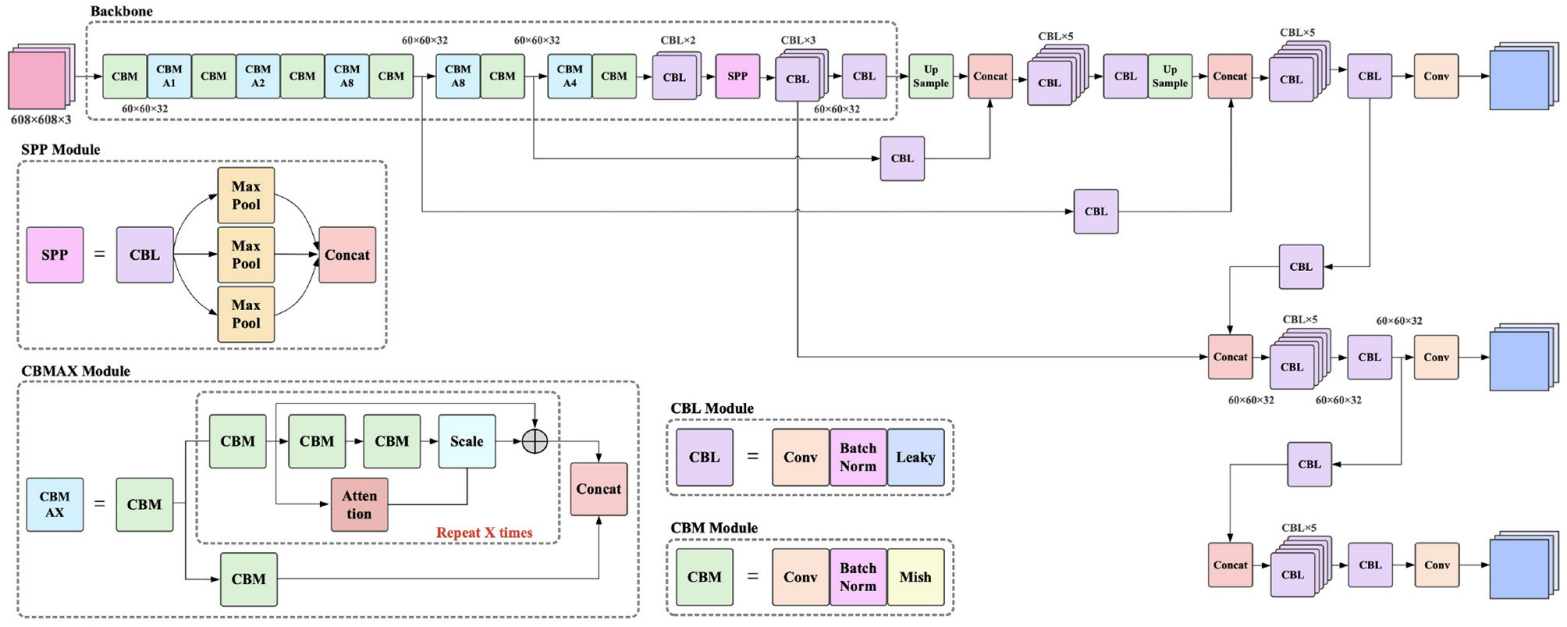
Illustration of our model.

The improved network result is shown in Figure 5.

#### 2.2.1. Attention Refinement Module

Human vision's visual attention system is a type of brain signal processing mechanism. Human vision can scan and understand an image quickly and select the object area that requires attention. Subsequently, greater focus is placed on this area in order to collect more details about the subject that needs attention while suppressing irrelevant data. In computer vision, this attention method is also commonly utilized. An autonomous driving business, Momenta, introduced a new image detection framework in 2017 that models the connection between feature channels and employs attention processes to improve the accuracy of critical features (Hu et al., 2018).

Our wheat head detection model employed the attention refinement module for the context information branch to refine the output of the last two stages, inspired by the successful use of attention mechanisms in autonomous driving. To integrate the semantic information of the global context, the global average pooling (Lin et al., 2013) approach was first employed to determine the greatest receptive field. The attention module training network then learned the characteristics with varying weights in the following steps. The attention refining module calculated the weight of each channel in the feature map. It then weighted each original output channel with the relevant weight to create a new weighted feature that may be used to further alter and integrate features. With only a tiny amount of computation, this attention method refined and optimized the output of two stages in the context information branch [specifically, the fourth and fifth stages of ResNet18 (He et al., 2016) down-sampling] and retrieves the global context semantic information fast and easily.

As shown in Figure 5, a generator was added inside “Attention.” Considering that InstanceNorm works better in generative tasks, all BatchNorm layers inside the generator were replaced with InstanceNorm. CGAN, CVAE, and CVAE-GAN were compared as generator models for “Attention,” and finally CVAE-GAN was chosen. Another change is that the common deconvolution upsampling and linear interpolation upsampling were replaced with the BiCubic interpolation upsampling algorithm, which has a better interpolation effect, as shown in Equations (1) and (2).


(1) $$W(x)=\left\{ \begin{array}{l} \;(a+2)|x{|}^{3}-(a+3)|x{|}^{2}+1,|x|\leq 1\\ a|x{|}^{3}-5a|x{|}^{2}+8a|x|-4a,1<|x|<2\\ \;0,otherwise \end{array}\right.$$

(2) $$B(x,y)=\sum_{i=0}^{3}\sum_{j=0}^{3}a_{ij}\times W(i)\times W(j)$$

In this case, calculating the coefficients *a*_*ij*_ depends on the properties of the interpolated data. If the derivatives of the interpolation function are known, a standard method is to use the heights of the four vertices and the three derivatives of each vertex. The first derivatives *h*′*x* and *h*′*y* represent the surface slope in the *x* and *y* directions, respectively. The second mutual derivative *h*″*xy* represents the slope in both the *x* and *y* directions. These values can be obtained by successively differentiating the *x* and *y* vectors, respectively. For each vertex of the grid cell, the local coordinates (0, 0), (1, 0), (0, 1), (1, 1) are substituted into these equations.

The main function of the generator is to generate a noise mask based on the attention feature maps extracted by the backbone of the main detection network and attention extractor, and to improve the feature learning ability of the main detection network by adding noise to the attention feature maps. Its role is similar to the dropout function in classification CNNs.

#### 2.2.2. Feature Fusion Module

The fusion of characteristics from multiple scales is a critical way to improve segmentation performance in many jobs. Low-level features have a better resolution and contain more location and detail information, but they have less convolution; as a result, they have worse semantics and more noise. High-level characteristics include more important semantic information, but they have limited resolution and poor detail perception. Improving the segmentation model requires effectively combining them.

The features obtained by the spatial information branch of the model in this article comprised a wealth of image space details. The features acquired by the context information branch, on the other hand, provided a wealth of image context information. The two models' output features were not on the same level, one being deep and the other shallow. As a result, merging them directly proved unfeasible, and a fusion module was required to complete the fusion of these features at various scales. To select and combine features, an FFM learning attentional mask was utilized. To achieve feature fusion, the steps listed below were used. (1) The traditional convolution operation was undertaken in the fusion module after a concatenated series is directly employed for diverse input characteristics. (2) The attention method utilized by the SENet (Hu et al., 2018) model was followed for feature optimization. (3) Using global average pooling, feature vectors for series features were produced. (4) Using convolution and activation functions, the weights of distinct features were determined. (5) The re-weighted features were multiplied by the features and weights, then added to the original features.

#### 2.2.3. Loss Function

The loss function of our model consists of three parts: box coordinate error, *CIoU* error, and classification error [see Equations (3)–(6)]. Box coordinate error (*x*_*i*_, *y*_*i*_) is the center position coordinate of the predicted box, and (*w*_*i*_, *h*_*i*_) is the width and height of the predicted box. Correspondingly, $\left(\hat{x_{i}},\hat{y_{i}}\right)$ and $\left(\hat{w_{i}},\hat{h_{i}}\right)$ are the labeled ground truth box coordinates and size. Additionally, λ_*coord*_ and λ_*noobj*_ are constants; *K* × *K* is the number of grids; *M* is the total number of predicted boxes; and $I_{ij}^{obj}$ is 1 when the *ith* grid contains a detection target and 0 in other cases.


(3) $$\begin{array}{l} Loss=Loss_{bounding\text{\_}box}+Loss_{ciou}+Loss_{classification} \end{array}$$

(4) $$\begin{array}{l} Loss_{bounding\_box}=\lambda_{coord}\sum_{i=0}^{K\times K}\sum_{j=0}^{M}I_{ij}^{obj}(2-w_{i}\times h_{i})[{\left(x_{i}-{\hat{x}}_{i}\right)}^{2}\\ \;+{\left(y_{i}-{\hat{y}}_{i}\right)}^{2}]+\\ \;\lambda_{coord}\sum_{i=0}^{K\times K}\sum_{j=0}^{M}I_{ij}^{obj}(2-w_{i}\times h_{i})[{\left(w_{i}-{\hat{w}}_{i}\right)}^{2}\\ \;+{\left(h_{i}-{\hat{h}}_{i}\right)}^{2}] \end{array}$$

(5) $$\begin{array}{l} Loss_{ciou}=\sum_{i=0}^{K\times K}\sum_{j=0}^{M}I_{ij}^{obj}[{\hat{C}}_{i}log(C_{i})+(1-{\hat{C}}_{i}log(1-C_{i})]+\\ \;\lambda_{noobj}\sum_{i=0}^{K\times K}\sum_{j=0}^{M}I_{ij}^{noobj}[{\hat{C}}_{i}log(C_{i})+(1-{\hat{C}}_{i}log(1-C_{i})] \end{array}$$

(6) $$\begin{array}{l} Loss_{classification}=\sum_{i=0}^{K\times K}I_{ij}^{obj}\hat{p}\sum_{c\in classes}[{\hat{p}}_{i}(c)\text{log}(p_{i}(c))\\ \text{ +(1}-{\hat{p}}_{i}(c)\text{log}(1-p_{i}(c))] \end{array}$$

Zheng et al. (2020) proposed a more effective *IoU* calculation method, *CIoU*, whose formula is Equation (7).


(7) $$\begin{array}{l} CIoU=1-IoU+\frac{\rho^{2}\left(A,B\right)}{c^{2}}+\alpha \nu \end{array}$$

The categories of classification are defined in the model as two categories, namely, positive and negative. For each ground truth box, the prediction box and its *IoU* are calculated. The largest *IoU* is a positive class, and the others are negative classes.

#### 2.2.4. Label Smoothing

Machine learning samples usually have a small number of mislabels, affecting the prediction performance. Label smoothing solves this problem by assuming that the labels may be incorrect at training time and avoiding “overconfidence” in the labels of the training samples. When the objective function is cross-entropy, a straightforward implementation of this idea is labeled smoothing.

In each iteration, instead of putting (*x*_*i*_, *y*_*i*_) directly into the training set, an error rate ϵ is set, and (*x*_*i*_, *y*_*i*_) is substituted into the training with probability 1-ϵ, and (*x*_*i*_, 1−*y*_*i*_) is substituted into the training with probability ϵ. In this way, the model is trained with both correct and incorrect label inputs, and it is conceivable that the model so trained will not match every label “to the fullest extent”, but only to a certain extent. In this case, the model will be less affected if there are indeed incorrect labels.

When using cross-entropy to describe the loss function, for each sample *i*, the expression of the loss function is:


(8) $$\begin{array}{l} L_{i}=-y_{i}P\left(\hat{y_{i}}=1|x_{i}\right)-\left(1-y_{i}\right)P\left(\hat{y_{i}}=0|x_{i}\right) \end{array}$$

After randomization, the new labels have the same probability of 1-ϵ as *y*_*i*_ and a different probability of epsilon (i.e., 1-*y*_*i*_). Therefore, when the randomized labels are used as training data, the loss function has the same probability of 1-ϵ as the above equation, and the probability of having ϵ is as Equation (9) shown:


(9) $$\begin{array}{l} L_{i}=-\left(1-y_{i}\right)P\left(\hat{y_{i}}=1|x_{i}\right)-y_{i}P\left(\hat{y_{i}}=0|x_{i}\right) \end{array}$$

By taking the above two equations as a probability-weighted average, we get Equation (10):


(10) $$\begin{array}{l} L_{i}=-\left[\epsilon \left(1-y_{i}\right)+\left(1-\epsilon \right)y_{i}\right]P\left(\hat{y_{i}}=1|x_{i}\right)\\ -\left[\epsilon y_{i}+\left(1-\epsilon \right)\left(1-y_{i}\right)\right]P\left(\hat{y_{i}}=0|x_{i}\right) \end{array}$$

Let $y_{i}^{\prime }=\epsilon \left(1-y_{i}\right)+\left(1-\epsilon \right)y_{i}$, we can simplify the above Equation (10): to get Equation (11):


(11) $$\begin{array}{l} L_{i}=-y_{i}^{\prime }P\left(\hat{y_{i}}=1|x_{i}\right)-\left(1-y_{i}^{\prime }\right)P\left(\hat{y_{i}}=0|x_{i}\right) \end{array}$$

Compared with the original cross-entropy Equation (8), only *y*_*i*_ is replaced with $y_{i}^{\prime }$ in Equation (11), and all other contents remain unchanged. This is logically equivalent to replacing each label *y*_*i*_ with $y_{i}^{\prime }$ and then performing the normal training process. Therefore, we do not need to randomize before training, but just replace each label.

#### 2.2.5. Optimization for NMS

In the classical object detection algorithm, in order to improve the Recall rate for the target, a dense number of anchor boxes are generated in the anchor phase. Therefore, there are many redundant frames corresponding to the same target during post-processing. Therefore, NMS is an essential step in removing the redundant boxes in post-processing. However, it has the following drawbacks:

1. Object overlap: as in the first figure below, there will be a box with the highest score. If NMS is adopted, we will delete the other prediction box with a slightly lower confidence level, which represents another object (due to overlap with the box with the highest confidence level being too large).
2. There are some cases that all boxes are not predicted correctly or not all boxes are accurate. Sometimes there are even cases where all the boxes around an object are labeled, but they are still not accurate.
3. The traditional NMS method is based on classification scores, and only the predicted boxes with the highest scores can remain. Nevertheless, in most cases, IoU and classification scores are not strongly correlated, and the positions of many boxes with high confidence in classification labels are not very accurate.

Therefore, this paper introduced soft NMS, the core of which is not to remove the redundant detection directly by an NMS threshold but to suppress the highly redundant detection results by a penalty function so that its score decreases. To be more specific, the more redundant the IOU is, the more its score decreases.

Both NMS and soft NMS exclude some boxes, while weighted boxes fusion (WBF) uses all boxes. Therefore, it can fix the case that all models are predicted inaccurately. Moreover, WBF will use all the predicted frames to fuse it. Therefore, this paper also uses WBF for experimental comparison.

## 3. Experiment

Our model was compared against other one-stage models such as EfficientDet (Tan et al., 2020) and the YOLO series models [YOLOv3 (Redmon and Farhadi, 2018), YOLOv4 (Bochkovskiy et al., 2020), and YOLOv5] in an experiment. Simultaneously, comparative experiments with the popular two-stage models were carried out. Our model was trained using the warm-up approach. The pseudo label method was used to fully utilize validation set data to improve the training process. The model fusion method was utilized to further increase the accuracy of the findings. An ablation experiment was undertaken to determine which strategies were most effective in enhancing the accuracy of the results.

### 3.1. Warm-Up

**Figure 6:**
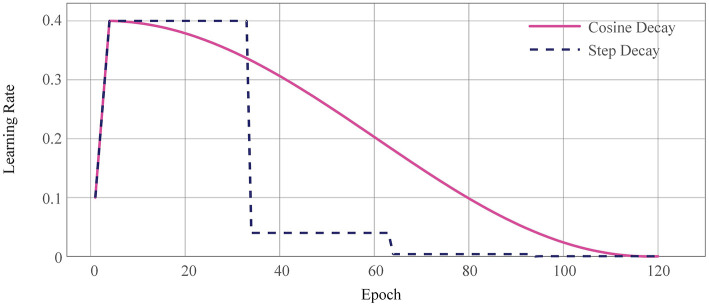
Warm-up learning rate curve. The dashed line corresponds to the segmented constant learning rate; the red curve is the warm-up learning rate decay strategy used in this paper.

In deep learning tasks such as object detection, the model is usually warmed up (He et al., 2016) first instead of using a linear learning rate tuning strategy. That is to say, it is gradually increased to a set learning rate with a small learning rate, which will lead to better final convergence. The warm-up technique is generally used in papers and competitions, especially in tasks where the model is difficult to converge. In this paper, we used two learning rate adjustment strategies, MultiStepLR and CosineAnnealingLR, whose learning rate variation curves are shown in Figure 6.

### 3.2. Pseudo Label

As a semi-supervised learning method, the pseudo-labeling technique plays an important role when the training dataset is insufficient. This paper uses a small training dataset and a large test dataset. The small training set is likely to lead to overfitting of the model, and the pseudo-labeling technique can be used to label the test set data to achieve a rapid augmentation of the training set.

There is a big difference between object detection and classification tasks in the production of pseudo labels. In the production of pseudo labels for classification tasks, there is only one label for a picture (e.g., there is only one positive and negative label for a picture in a binary classification task). Therefore, we only need to select samples with high prediction probability (select samples with prediction probability greater than 0.99 and label them as 1) to train the model as positive samples. However, in the object detection task, a picture has multiple labels, if only the labels with high prediction probability are selected, then there will be many wheat heads in a picture that are not labeled as negative samples, which will lead to a decrease in the detection ability of the model. So the prediction probability threshold in the target detection task becomes a key to the pseudo labeling, which cannot be too high but at the same time cannot be too low (too low will introduce some wrong labels). Our solution is to use a sliding threshold to search for the best threshold of the model first and then fine-tune this threshold as the threshold for making a pseudo label, which is generally low, namely 0.15.

**Figure 7:**
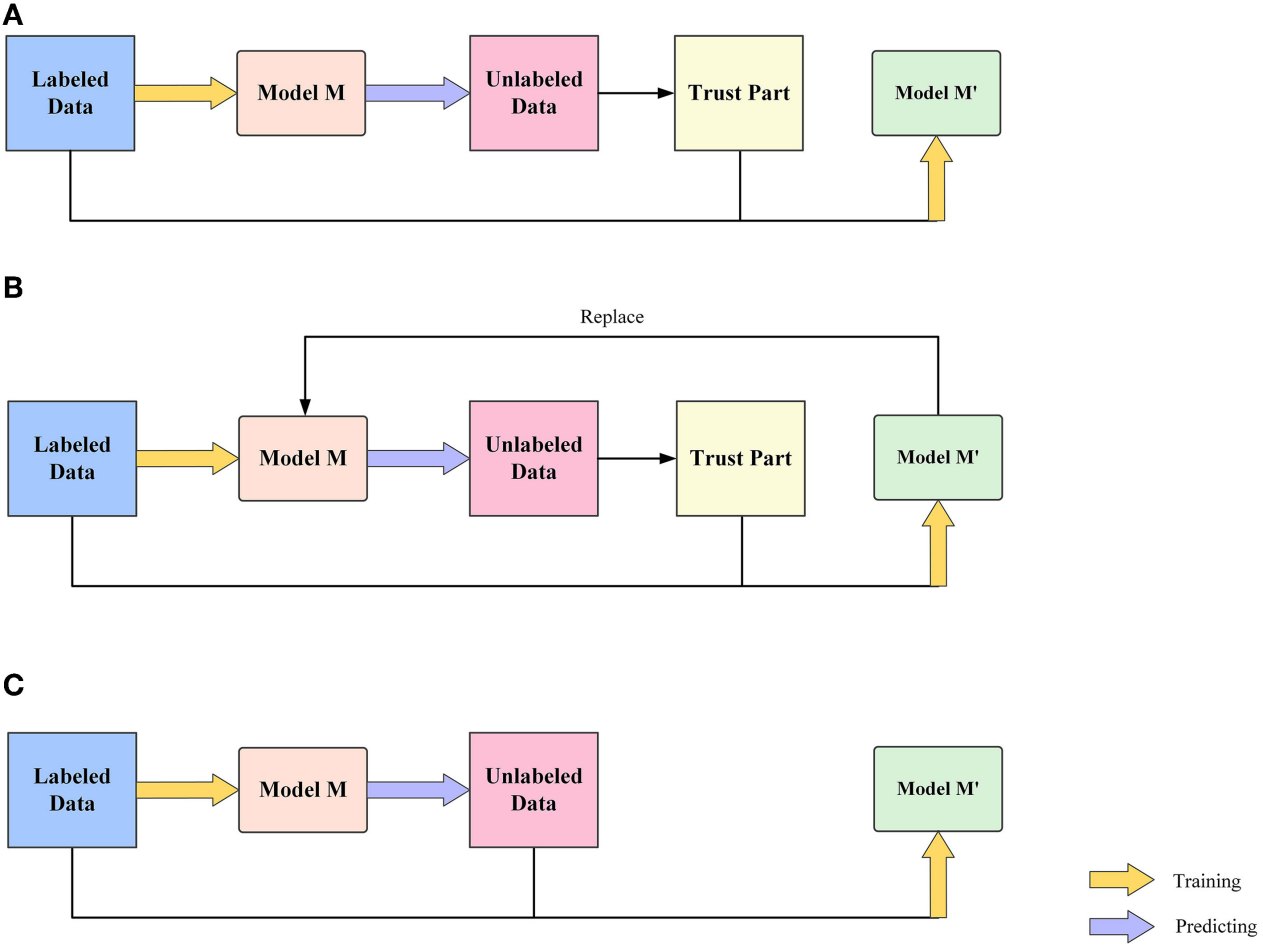
Flow chart of three pseudo-label models: **(A)** Model include the step of placing the model prediction labels into the Trust Part, **(B)** Model include the step of placing the prediction labels intro the Trust Part, also with iterative process, and **(C)** Model without the step of placing the model prediction labels into the Trust Part.

We have three different implementations of the pseudo label method, as shown in Figure 7.

### 3.3. Experiment Result

Our model and seven other methods were used to detect wheat heads under the same experimental conditions, and the mAP and FPS values of the models were compared. The results are shown in Table 1.

**Table 1:** Comparison of variant models and our model.

**Method**	**mAP**	**FPS**	**Batch size**	**Input resolution**
FasterRCNN	0.5396	7	2	600 × 600
MaskRCNN	0.6493	9	2	600 × 600
EfficientDet	0.6520	107	8	512 × 512
YOLOv3	0.5806	23	2	608 × 608
YOLOv4	0.6385	127	2	608 × 608
YOLOv5	0.6674	**151**	2	608 × 608
SSD300	0.6465	45	2	300 × 300
SSD300	0.6460	62	8	300 × 300
SSD512	0.6477	19	2	512 × 512
SSD512	0.6478	21	8	512 × 512
Our model 512	0.6825	147	2	512 × 512
Our model 512	**0.6893**	**151**	8	512 × 512
Our model 1024	0.6756	79	2	1024 × 1024

*The bold values were used to label the best performing score compared with others*.

The model fusion method was then used to improve mAP. The model fusion method is simple because it calculates the intersection of the results of multiple models directly. In this study, the model fusion method was used to combine our model, FasterRCNN, and YOLO models, as shown in Table 2.

**Table 2:** Results of model fusion.

**Models**	**OoF**	**NMS method**	**mAP**
Our model 512		NMS	0.6847
Our model 1024		NMS	0.6756
Our model 512	+	soft NMS	0.6893
Our model 512 + YOLO series + MaskRCNN	+	WBF	**0.6991**

*The bold values were used to label the best performing score compared with others*.

The experimental results show that the mAP obtained when fusing the our model512 + YOLO series(v3 + v4 + v5) + MaskRCNN models is 0.6991, which is already higher than that of Leaderboard #1 in Global Wheat Detection (mAP: 0.6897). It must be noted that although this competition does not allow participants to use the YOLO-v5 model because YOLO-v5 does not comply with the MIT License. However, in previous experiments, even using YOLO-v5 alone, the mAP only reached 0.6674.

### 3.4. Ablation Experiments of Generative Methods

This paper uses three generative models to optimize the attention module: CGAN, CVAE, and CVAE-GAN. To verify their respective implementation effects, ablation experiments are carried out in this paper. Table 3 illustrates the experimental results.

**Table 3:** Comparison of variant models and our model.

**Model**	**Attention module**	**mAP**	**FPS**	**Batch size**	**Input resolution**
YOLOv5	Baseline	0.6674	**151**	2	608 × 608
Our model	CGAN	0.6791	116	2	512 × 512
Our model	CVAE	0.6825	147	2	512 × 512
Our model	CVAE-GAN	0.6837	108	2	512 × 512

*The bold values were used to label the best performing score compared with others*.

From the Table 3, it can be seen that CVAE-GAN combines the advantages of CGAN and CVAE, respectively. However, this model inference speed is also the slowest. By comparing the baseline model, we can find that various optimization of the attention module effectively promote our model's performance.

### 3.5. Result Analysis

To verify the effectiveness of the various pre-processing techniques proposed in this study, ablation experiments were performed on both our model512 and model1024. The experimental results are shown in Tables 4, 5.

**Table 4:** Ablation experiment results on our model512.

**Cutout**	**CutMix**	**Mosiac**	**Warm-up**	**Label smoothing**	**Pseudo label**	**mAP**
			+	+	A	0.5020
+	+	+	+	+	C	**0.6893**
+		+	+	+	A	0.6735
+	+	+	+	+	B	0.6770
+	+		+	+	C	0.6708
+		+	+		C	0.6681
+		+		+	C	0.6595

*The bold values were used to label the best performing score compared with others*.

**Table 5:** Ablation experiment results on our model1024.

**Cutout**	**CutMix**	**Mosiac**	**Warm-up**	**Label smoothing**	**Pseudo label**	**mAP**
+	+	+	+	+	C	0.6687
+		+	+	+	A	**0.6756**
+	+	+	+	+	B	0.6741
+		+	+		B	0.6713
+		+		+	C	0.6520

*The bold values were used to label the best performing score compared with others*.

Through the analysis of experimental results, it was found that data enhancement methods such as Cutout, CutMix, and Mosaic greatly improved the performance of our model. The principles of CutMix and Mosaic are similar, and it was found that compared with adopting both methods, using CutMix or Mosaic alone exerted a more significant effect on improving model performance. It was also found that the model performed best when warm-up, label smoothing, and pseudo label methods were used simultaneously.

### 3.6. Software Design

**Figure 8:**
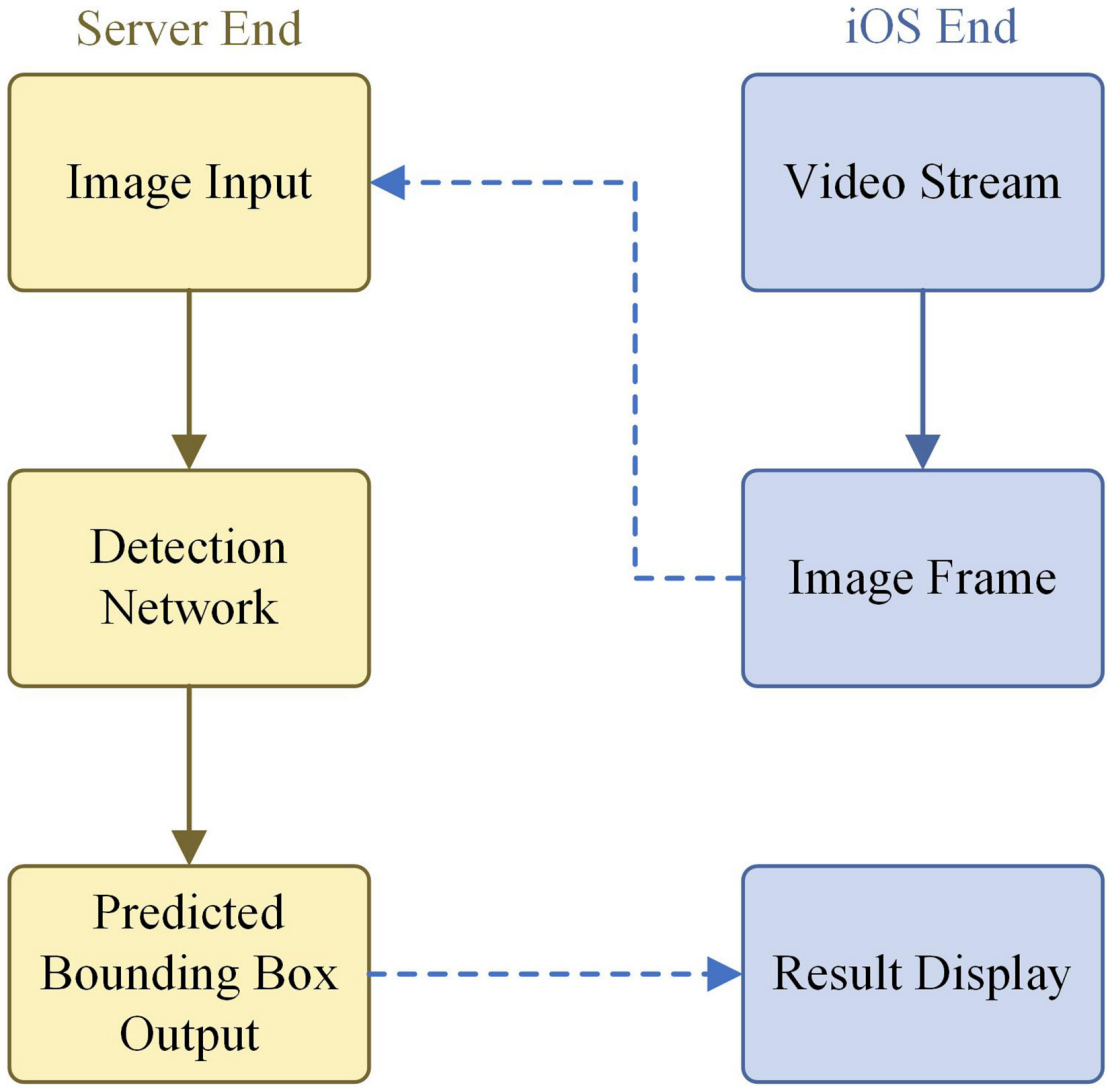
Intelligent wheat detection system flowchart.

In order to realize the end-to-end model of wheat detection and promote the efficiency of recognizing and labeling, an intelligent diagnosis system based on our model was developed as an app for iOS using the programming language Swift. The workflow of the system is shown in Figure 8.

**Figure 9:**
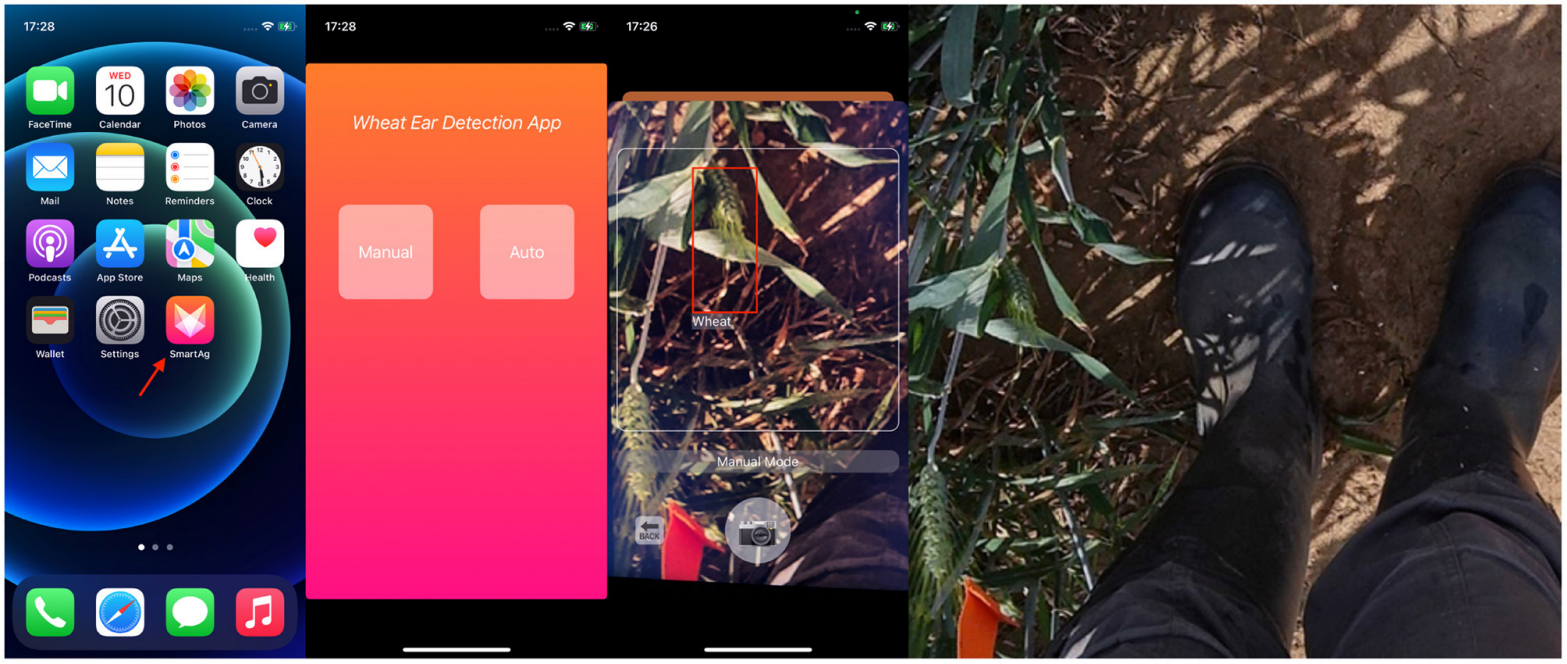
Screenshot on iPhone12 mini. From left to right: screenshot of the app launched on the desktop; screenshot of the function selection after launching; screenshot of the test result; and image of wheat used for testing.

The app works as follows. First, a video stream of wheat is accessed *via* the iPhone's camera. Then the representative frames are extracted and sent to the server. Next, the server transfers the received images to the trained our model. Finally, the output of the model is returned to the iOS end, and the iOS end draws a detection frame based on the returned parameters. Some screenshots of the app in action are shown in Figure 9. The app has been submitted to Apple's App Store.

Two functional modes were created for this app. The manual mode requires the user to take a picture manually for detection. The automatic mode takes a frame from the video stream every second for automatic detection and result archives.

## 4. Conclusions

This study suggested a novel wheat head detection model based on the widely used single-stage object detection network model, YOLO, with the purpose of detecting wheat quickly. The main innovation of the network model proposed in this paper can be summarized in the following points: (1) Add generative sub-network to the attention module to improve the main detection network's performance; (2) Replace the NMS algorithm in the detection network with WBF algorithm; (3) Replace the original *GIoU* calculation in the network by introducing *CIoU* to the loss function. Adding an attention mechanism and a multi-scale feature fusion module, as well as improving the activation function, increased the model's performance. Data augmentation methods containing Cutout, CutMix, and Mosaic, as well as technical methods like label smoothing and pseudo label, were used to make the most of the training data set and expand the training data samples. The model's effect was optimized *via* test time augmentation, OoF, WBF, and model fusion. Comparative and ablation experiments were carried out to verify the model's efficiency. According to the findings, the proposed wheat head detection network's inference time could approach 25 ms, and an *mAP* of 0.688 was realized for wheat head detection. A mobile software based on Swift and PHP was built to allow this network to be applied on iOS mobile terminals, allowing it to be widely used in the agricultural production scenario.

## Data Availability Statement

The original contributions presented in the study are included in the article/supplementary material, further inquiries can be directed to the corresponding author/s.

## Author Contributions

YZ: conceptualization and methodology. YZ and ML: validation. YZ, ML, and YW: writing—original draft preparation. YZ, ML, XM, XW, and YW: writing—review and editing. YZ and XM: visualization. YW: supervision and funding acquisition. All authors have read and agreed to the published version of the manuscript.

## Conflict of Interest

The authors declare that the research was conducted in the absence of any commercial or financial relationships that could be construed as a potential conflict of interest.

## Publisher's Note

All claims expressed in this article are solely those of the authors and do not necessarily represent those of their affiliated organizations, or those of the publisher, the editors and the reviewers. Any product that may be evaluated in this article, or claim that may be made by its manufacturer, is not guaranteed or endorsed by the publisher.
